# Short-Term HRV Analysis Using Nonparametric Sample Entropy for Obstructive Sleep Apnea

**DOI:** 10.3390/e23030267

**Published:** 2021-02-24

**Authors:** Duan Liang, Shan Wu, Lan Tang, Kaicheng Feng, Guanzheng Liu

**Affiliations:** 1 School of Biomedical Engineering, Sun Yat-Sen University, Guangzhou 510275, China; liangd35@mail2.sysu.edu.cn (D.L.); wush69@mail2.sysu.edu.cn (S.W.); tanglan@mail2.sysu.edu.cn (L.T.); fengkch@mail2.sysu.edu.cn (K.F.); 2 Key Laboratory of Sensing Technology and Biomedical Instruments of Guangdong Province, School of Engineering, Sun Yat-Sen University, Guangzhou 510275, China; 3 Guangdong Provincial Engineering and Technology Centre of Advanced and Portable Medical Device, Guangzhou 510006, China

**Keywords:** heart rate variability (HRV), nonparametric sample entropy (NPSampEn), obstructive sleep apnea (OSA), short-term HRV analysis

## Abstract

Obstructive sleep apnea (OSA) is associated with reduced heart rate variability (HRV) and autonomic nervous system dysfunction. Sample entropy (SampEn) is commonly used for regularity analysis. However, it has limitations in processing short-term segments of HRV signals due to the extreme dependence of its functional parameters. We used the nonparametric sample entropy (NPSampEn) as a novel index for short-term HRV analysis in the case of OSA. The manuscript included 60 6-h electrocardiogram recordings (20 healthy, 14 mild-moderate OSA, and 26 severe OSA) from the PhysioNet database. The NPSampEn value was compared with the SampEn value and frequency domain indices. The empirical results showed that NPSampEn could better differentiate the three groups (*p* < 0.01) than the ratio of low frequency power to high frequency power (LF/HF) and SampEn. Moreover, NPSampEn (83.3%) approached a higher OSA screening accuracy than the LF/HF (73.3%) and SampEn (68.3%). Compared with SampEn (|r| = 0.602, *p* < 0.05), NPSampEn (|r| = 0.756, *p* < 0.05) had a significantly stronger association with the apnea-hypopnea index (AHI). Hence, NPSampEn can fully overcome the influence of individual differences that are prevalent in biomedical signal processing, and might be useful in processing short-term segments of HRV signal.

## 1. Introduction

Obstructive sleep apnea (OSA) is the most common type of apnea, which is characterized by repetitive collapse or partial collapse of the pharyngeal airway during sleep [1]. OSA is common in patients with high blood pressure, coronary artery disease, stroke, and atrial fibrillation [2,3]. The primary pathophysiological mechanism is cardiovascular autonomic dysfunction, which mainly manifests as sympathetic overactivation [4]. The prevalence of OSA is 4% in men and 2% in women at the rate of 10 events per hour, but more than 85% of OSA patients never receive a definite diagnosis [5,6,7].

Changes of respiration and blood pressure affect the balance of the autonomic nervous system (ANS), thus affecting heart rate variability (HRV) [8]. The ANS modulates physiological function through the sympathetic nervous system (SNS) and parasympathetic nervous system (PNS) [9]. The parameters of HRV have been proven to be significant indices for analyzing and diagnosing OSA [10]. Traditionally, linear methods such as time and frequency domain analyses have been used to analyze OSA. The ratio of low-frequency to high-frequency power (LF/HF) is the classic frequency domain index, which is considered to reflect the balance of the ANS. A higher LF/HF indicates that the sympathetic nerve is more active, and a lower LF/HF indicates that the parasympathetic nerve is more active [11]. Gula, L.J. et al. found that the value of LF/HF was higher in patients with moderate OSA than that in healthy subjects and severe OSA patients, and Gong, X. et al. found that the value of the apnea-hypopnea index (AHI) was positively correlated with the LF/HF [12,13]. These results suggest that the LF/HF is effective in discriminating individuals with and without OSA.

The autonomic nervous system is nonlinear and nonstationary [14,15]; thus, nonlinear methods are widely used for OSA detection. Entropy is considered as a nonlinear method that is used to analyze the complexity of ANS. Sample entropy (SampEn) analysis has been used to show that the HRV in normal individuals is significantly more complex than that in individuals with OSA (*p* < 0.005) by Al-Angari, H.M. et al. [16]. Ravelo-Garcia, A. G et al. used permutation entropy to better detect OSA with HRV analysis [17]. Sliding trend fuzzy approximate entropy has been proposed for use in HRV analysis, and demonstrated high accuracy (85%) with a sensitivity of 82.5% and a specificity of 90% for OSA detection [18].

For HRV analysis, most of the previous studies used a 5-min segment of electrocardiogram (ECG) ECG signal to assess the ANS [12,19]. Nevertheless, reference annotations were typically created for every minute with 60–100 R peak to R peak (RR) RR intervals to indicate the presence or absence of apnea during that minute [20,21]. Furthermore, SampEn is highly dependent on parameter r, which is the threshold of distance. SampEn is not suitable for short-term HRV analysis because an improper r value may cause the SampEn value to be undefined [22]. Several studies have also reported that inappropriate r was randomly selected in the SampEn calculation, which led to misleading regular results [23,24]. Therefore, in this study, a method based on non-parametric sample entropy (NPSampEn) is proposed to reflect the influence of self-fluctuation and overcome individual differences, thus improving the performance of OSA screening and quantification.

For NPSampEn, the r sequence was generated based on the unique template distance of the signal. The distribution of the template distance was estimated by r sequence to evaluate the regularity of the signal and the fluctuation of the autonomic nerve. The process was as follows. First, the distance matrix of 1-minute RR segments was calculated as in the SampEn analysis. Then, the extracted sequence containing unique points from the distance matrix was used as the packet interval to calculate the cumulative probability and identify abnormal sympathetic signal variance. Finally, the NPSampEn was used to detect sleep apnea and quantify the severity of OSA disease.

## 2. Materials and Methods

**Figure 1:**
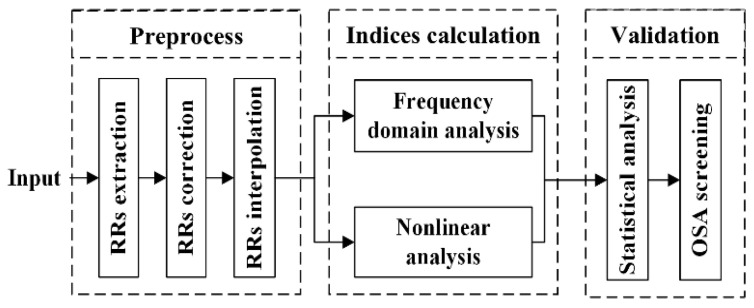
Framework of the proposed method of analyzing heart rate variability (HRV).

In our study, a new nonlinear analysis method was used for short-term HRV analysis. The framework of the study is shown in Figure 1. First, the ECG signals were downloaded from the database, and the RR intervals were extracted. Then, the corrected 1-min RR segments were obtained and resampled to 2 Hz by interpolation. Then, short-term HRV measures and the NPSampEn were used to determine the indices in the frequency domain and nonlinear analyses. Finally, these short-term indices were validated by statistical analysis and OSA screening.

### 2.1. Data

To develop and evaluate our HRV-based algorithm, we used the Computers in Cardiology Challenge 2000 of the PhysioNet database [21]. It contains 70 single channel overnight ECG recordings from 32 subjects. The dataset provided a minute-by-minute sequence of reference annotations for the ECG recordings. Based on the apnea-hypopnea index (AHI), which was calculated by determining the sum of apneas and hypopnea events per hour during sleep [25], all recordings were divided into three groups. Recordings with a 1-hour AHI of 10 or more were included in the apnea group. There were 40 recordings in the apnea group with AHI ≥ 5. The subjects were 15 males and one female between 29 and 63 years of age (50 ± 6.6 years). Recordings with five minutes or less of sleep apnea were included in the normal group. There were 20 recordings in the normal group with AHI < 5. The subjects were six males and five females between 27 and 42 years of age (33 ± 5.8 years). In addition, the remaining recordings were included in the borderline group. In this study, we used the normal group and apnea group to develop and evaluate the algorithm.

To analyze the severity of OSA disease, according to the study of Li et al. [26], the apnea group was further divided into a mild-moderate OSA group and a severe OSA group through AHI. The mild-moderate OSA group included 14 recordings with 5 ≤ AHI < 30. The severe OSA group included 26 recordings with AHI ≥ 30.

### 2.2. Simulation Test

A simulation signal was created to compare the performance of SampEn and NPSampEn in the short-term HRV analysis. The N-point simulation signal *MIX* (*p*) was defined as follows [27]:

(1) $$MIX{\left(p\right)}_{j}=\left(1-Z_{j}\right)X_{j}+Z_{j}Y_{j}\;\left(1\leq j\leq N\right),$$

where $X_{j}=\sqrt{2}\sin\left(\frac{2\pi j}{12}\right)$;$\;Y_{j}$ was a set of real random variables within $\left[-\sqrt{3},\sqrt{3}\right]$, and $Z_{j}$ was a vector related to *p*, which consisted of 0 and 1, where $Z_{j}=1$ had a probability of *p* and $Z_{j}=0$ had a probability of *1-p*. Simply, when p=0, $Z_{j}$ fully consisted of 0; when p=1, $Z_{j}$ fully consisted of 1. Hence, the value of *p* reflected the complexity of the MIX(p) signals. As the *p* value increased, the random components and complexity of MIX(p) increased. To compare the performance of SampEn and NPSampEn, we calculated the SampEn and NPSampEn of the simulation signals with different lengths in the complexity of *MIX* (0), *MIX* (0.3), *MIX* (0.5).

### 2.3. Short-Term Heart Rate Variability (HRV) Analysis

#### 2.3.1. Preprocessing

To obtain appropriate RR segments for short-term HRV analysis, the ECG signals were preprocessed. The first six hours of overnight ECG signals were inputted. First, RR intervals were extracted from the ECG signals using the Pan-Tompkins algorithm and corrected using the local median filter proposed by Chen, L et al. [28,29]. Then, the 6-h RR intervals were divided into multiple nonoverlapping 1-min RR segments (RRs) in each recording for short-term HRV analysis. Finally, all 1-min RRs were resampled by 2 Hz with interpolation.

#### 2.3.2. Frequency Domain Analysis

There are three classic indices in short-term HRV analysis [11,12,13]: the power in the low-frequency range (LF, 0.04–0.15 Hz), the power in the high-frequency range (HF, 0.15–0.4 Hz), and the ratio of low-frequency and high-frequency power (LF/HF). The three frequency domain indices of 1-min RRs were calculated by power spectrum density (PSD), and the mean value in 6-h RRs was computed. Due to the short lengths of the RRs, the 6-order autoregressive Burg parametric method was used to calculate the PSD.

#### 2.3.3. Nonlinear Analysis

SampEn is a nonlinear method used to analyze the regularity of sequences by computing the similarity between template vectors. The signal vector was divided into a number of template vectors at the embedding dimensions *m* and *m* + 1, and the distance between the template vector and every other vector was computed. Finally, the tolerance parameter r (usually 0.1 to 0.25 times the standard deviation of the data [30]) was used as the threshold, and the probability of the template distance being less than the parameter ‘r’ was calculated to determine the similarity between template vectors. However, SampEn depends heavily on parameter ‘r’, and an incorrect r choice also leads to the opposite result. Hence, a SampEn measure independent of parameter ‘r’ was proposed by Udhayakumar, R.K. et al. [31].

**Figure 2:**
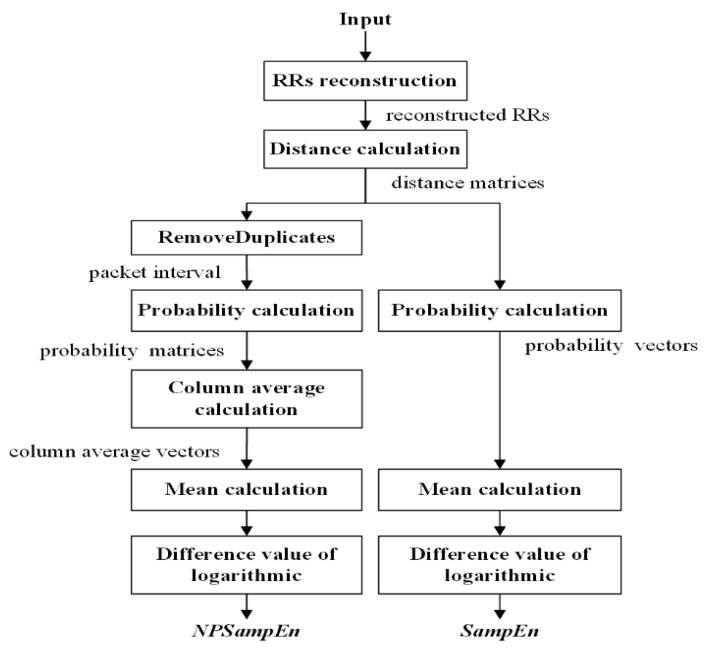
Nonlinear analysis method of NPSampEn and SampEn.

The core process of SampEn is to compare the template distance with the threshold ‘r’ to compute the probability, and NPSampEn is a measure that maintains this core process with improvements. For NPSampEn, the artificially selected parameter ‘r’ was not used as the threshold, instead, a tolerant vector from the signal itself was generated, and then, the probability was calculated based on the tolerant vector. The schemes of SampEn and NPSampEn are shown in Figure 2, and the algorithms of NPSampEn and SampEn are as follows:

For a time series with N data points, denoted as {x(n)|n=1,2,…,N}.

Step 1: RR reconstruction. The time series *x*(*n*) was divided into a number of template vectors at the embedding dimensions m and *m* + 1. When 1≤i≤N−m:

At the embedding dimension m,

(2) $$X_{i}^{m}=\left\{x\left(i\right),\ldots ,x\left(i+m-1\right)\right\}$$

Similarly, at the embedding dimension *m* + 1,

(3) $$X_{i}^{m+1}=\left\{x\left(i\right),\ldots ,x\left(i+m\right)\right\}$$

Step 2: Distance calculation. At dimensions m and *m* + 1, the distance between the template vector and every other vector was calculated.

(4) $$d_{ij}^{m}=max\left|x\left(i+k\right)-x\left(j+k\right)\right|$$

0≤k≤m−1,1≤j≤N−m and j≠i

Similarly,

(5) $$d_{ij}^{m+1}=max\left|x\left(i+k\right)-x\left(j+k\right)\right|$$

0≤k≤m,1≤j≤N−m and j≠i

Self-matches were excluded in both SampEn and NPSampEn, and two distance matrices, $d^{m}$ and $d^{m+1}$, were obtained with N−m dimensions without $d_{ij}^{m}$ and $d_{ij}^{m+1}$, when i=j.

Step 3: In the probability calculation, the NPSampEn value is different from the SampEn values.

For NPSampEn, let *scope* be the vector containing all unique elements of $d_{ij}^{m}$ and $d_{ij}^{m+1}$, sorted in ascending order with the length of *nbin*. The *pr* is the probability, *scope(q)* is the element of the *scope* vector (1≤q≤nbin), and the cumulative distribution function $pd_{i}^{m}$ is calculated as follows:

(6) $$pd_{i}^{m}\left(q\right)=pr\left(d_{i}^{m}\leq scope\left(q\right)\right),1\leq q\leq nbin$$

Similarly, at the embedding dimension *m* + 1,

(7) $$pd_{i}^{m+1}\left(q\right)=pr\left(d_{i}^{m+1}\leq scope\left(q\right)\right),1\leq q\leq nbin$$

The cumulative probability matrix is as follows:

(8) $$pd^{m}=\left[\begin{array}{ccc} pd_{1}^{m}\left(1\right) & \cdots & pd_{1}^{m}\left(nbin\right)\\ \vdots & \ddots & \vdots \\ pd_{N-m}^{m}\left(1\right) & \cdots & pd_{N-m}^{m}\left(nbin\right) \end{array}\right]$$

(9) $$pd^{m+1}=\left[\begin{array}{ccc} pd_{1}^{m+1}\left(1\right) & \cdots & pd_{1}^{m+1}\left(nbin\right)\\ \vdots & \ddots & \vdots \\ pd_{N-m}^{m+1}\left(1\right) & \cdots & pd_{N-m}^{m+1}\left(nbin\right) \end{array}\right]$$

For SampEn, the tolerance parameter *r* (usually 0.1 to 0.25 times the standard deviation of the data) was used as the threshold.

(10) $$pd_{i}^{m}=pr\left(d_{i}^{m}\leq r\right),1\leq i\leq N-m$$

(11) $$pd_{i}^{m+1}=pr\left(d_{i}^{m+1}\leq r\right),1\leq i\leq N-m$$

The probability vectors were obtained as follows:

(12) $$pd^{m}=\left[pd_{1}^{m},\ldots ,pd_{N-m}^{m}\right]$$

(13) $$pd^{m+1}=\left[pd_{1}^{m+1},\ldots ,pd_{N-m}^{m+1}\right]$$

Step 4: The values of NPSampEn and SampEn were calculated. We obtained the probability matrices for NPSampEn and the probability vectors for SampEn from step 3.

For NPSampEn, the vector $\Phi^{m}$ containing the column-average of probability was obtained.

(14) $$\Phi^{m}\left(q\right)=\frac{1}{N-m}\overset{N-m}{\underset{i=1}{\sum }}pd_{i}^{m}\left(q\right)$$

(15) $$\Phi^{m+1}\left(q\right)=\frac{1}{N-m}\overset{N-m}{\underset{i=1}{\sum }}pd_{i}^{m+1}\left(q\right)$$

The NPSampEn value was finally calculated

(16) $$NPSampEn=\frac{1}{nbin}\overset{nbin}{\underset{q=1}{\sum }}ln\frac{\Phi^{m}\left(q\right)}{\Phi^{m+1}\left(q\right)}$$

For SampEn, the mean of probability vector $\Phi^{m}$ was calculated.

(17) $$\Phi^{m}=\frac{1}{N-m}\overset{N-m}{\underset{i=1}{\sum }}pd_{i}^{m}$$

(18) $$\Phi^{m+1}=\frac{1}{N-m}\overset{N-m}{\underset{i=1}{\sum }}pd_{i}^{m+1}$$

The SampEn value was finally calculated

(19) $$SampEn=ln\frac{\Phi^{m}}{\Phi^{m+1}}$$

### 2.4. Validation

To evaluate the performance of a single index, statistical one-way ANOVA, followed by post-hoc analysis with the least significant difference (LSD) test was used to analyze the statistical significance between the normal, mild-moderate OSA, and severe OSA groups. *p* < 0.05 was considered statistically significant, and decreasing *p* values represented a tendency toward statistical significance. The data are expressed as the mean ± SD. Furthermore, support vector machine (SVM) in scikit-learn support of NuSVC class in Python 3 was used for classification, and the accuracy, sensitivity, and specificity were obtained using 2-fold cross validation [32]. Moreover, the receiver operating characteristic (ROC) curve and area under the ROC curve (AUC) of each index were computed using MATLAB. Finally, the Pearson coefficient of the association of SampEn and NPSampEn with AHI was computed using SPSS. These tests were performed using SPSS version 22.0.0.0 (SPSS Inc., Chicago, IL, USA) and MATLAB R2019a, Python 3.

## 3. Results

### 3.1. Comparisons between the Simulation of Two Entropies

**Figure 3:**
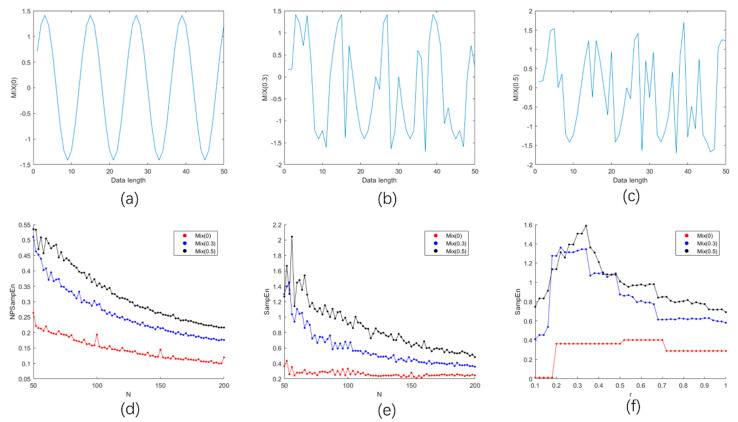
Fifty points simulated for the MIX (0), MIX (0.3), and MIX (0.5) signals (**a**–**c**). N corresponds to the data length, N increases from 50 to 200 by intervals of 2. The changes in NPSampEn (**d**), and SampEn (**e**) (r = 0.25). The change in SampEn (**f**) with an increase in r from 0.1 to 1 by 0.02 (N = 100) and the corresponding changes in the complexity of MIX (0), MIX (0.3), MIX (0.5).

To compare the performance between NPSampEn and SampEn, three simulated signals *MIX* (*p*) were constructed. Monotonicity, consistency, and continuity are three important factors that reflect the performance of entropies in complexity analysis [33]. Figure 3 indicates that entropies change with the length of signals. As shown in Figure 3d, the NPSampEn values increased with an increase in the complexity of the simulated signals, and decreased with the increase in the lengths of the simulated signals. There was no cross between the NPSampEn values for the three simulated signals. As illustrated in Figure 3e, there were crosses among the SampEn values between *MIX* (0.3) and *MIX* (0.5) when N was small (nearly 50), and there were fluctuations in the SampEn of *MIX* (0.3) and *MIX* (0.5) when N was small (N < 100). This result indicates that the value of SampEn was significantly affected by the length of the signal when the complexity of the signal was high. In Figure 3f, the increasing r value resulted in a tendency toward a lower SampEn value and represented high system regularity. The SampEn value of *MIX* (*p*) theoretically increased with the *p* value of the *MIX* (*p*) signal. However, the SampEn value of *MIX* (0.3) was sometimes higher than *MIX* (0.5). These results indicate that an improper r value can lead to the opposite conclusion.

### 3.2. HRV Analysis among the Three Groups

**Figure 4:**
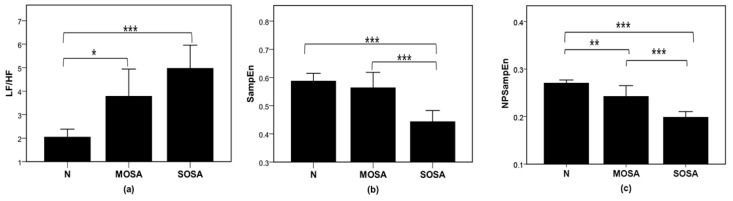
Comparison of the ratio of low frequency power to high frequency power (LF/HF) (**a**), SampEn (**b**), and NPSampEn (**c**) for the normal, mild-moderate OSA, and severe OSA groups. *, **, *** represent *p* < 0.05, *p* < 0.01, and *p* < 0.001, respectively

The recordings were divided into normal (N), mild-moderate OSA (MOSA), and severe OSA (SOSA) group. The mean ± SD values of the entropies and frequency domain indices are shown in Table 1. As shown in Table 1, in the frequency domain indices, there was no statistically significant difference in the LF or HF between any two of the three groups. The LF/HF was significantly different between the N and MOSA groups (*p* < 0.05) and N and SOSA groups (*p* < 0.001). Thus, the LF/HF can distinguish OSA patients and healthy persons, but cannot distinguish mild-moderate OSA patients and severe OSA patients. In the nonlinear indices, there were statistically significant differences in the N and SOSA groups and MOSA and SOSA groups. No significant differences were observed for SampEn between the N and MOSA groups, while NPSampEn was significantly different between the N and MOSA groups (*p* < 0.01). Figure 4 indicates that with the aggravation of pathogenetic conditions (from the N to MOSA to SOSA groups), the SampEn and NPSampEn values decreased, and the LF/HF value in the frequency domain increased. From a physiological point of view, the more severe the pathogenetic condition, the more complex HRV is, and the more active the sympathetic nerve is.

**Figure 5:**
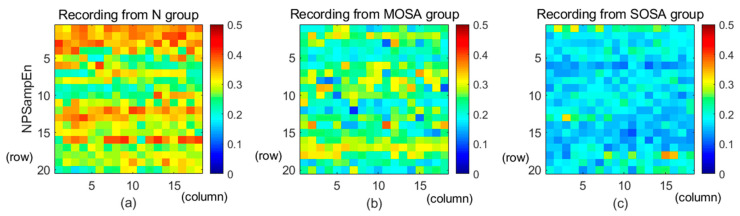
The NPSampEn values for three recordings. (**a**) NPSampEn values of a recording from the N group, (**b**) NPSampEn values of a recording from the mild-moderate OSA (MOSA) group, and (**c**) NPSampEn values of a recording from the severe OSA (SOSA) group.

As shown in Figure 5, the NPSampEn values of all RRs for three recordings from the three groups were rearranged into three matrices in order. Each number in the NPSampEn matrix represents the NPSampEn value computed from the RRs time series from a given recording. For example, the first row of the NPSampEn matrix includes the first to the 18th NPSampEn values, the second row includes the 19th to the 36th NPSampEn values, and so on. These matrices were made into graphs using MATLAB (R2019a, Mathworks, Natick, MA, USA) MATLAB. The color of each rectangle reflects the NPSampEn value of one minute. The normal group is represented by warm colors, while the severe OSA group is represented by cool colors. Therefore, based on three typical cases, the color became cooler with the severity of OSA. In short, NPSampEn is believed to be a more appropriate index for OSA detection and quantization.

### 3.3. Relevance and Obstructive Sleep Apnea (OSA) Screening

**Figure 6:**
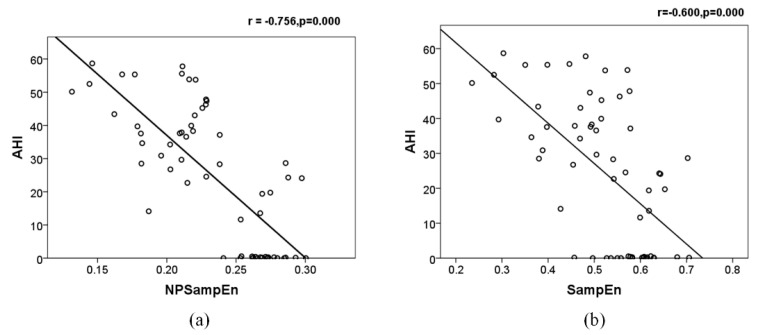
The relevance of NPSampEn (**a**) and SampEn (**b**) with apnea-hypopnea index (AHI). r: Pearson’s correlation coefficient; p: *p*-value.0.5 < |r| < 0.8 represent significant correlation.

Figure 6 indicates the correlation between AHI and NPSampEn and the correlation between AHI and SampEn. When it is significantly different (*p* < 0.05), the greater the absolute Pearson correlation (R) and the stronger the correlation. Negative R represents a negative correlation. The correlation between AHI and NPSampEn was strong (|r| = 0.756, *p* < 0.001), while the correlation between AHI and SampEn was weak (|r| = 0.600, *p* < 0.001).

**Figure 7:**
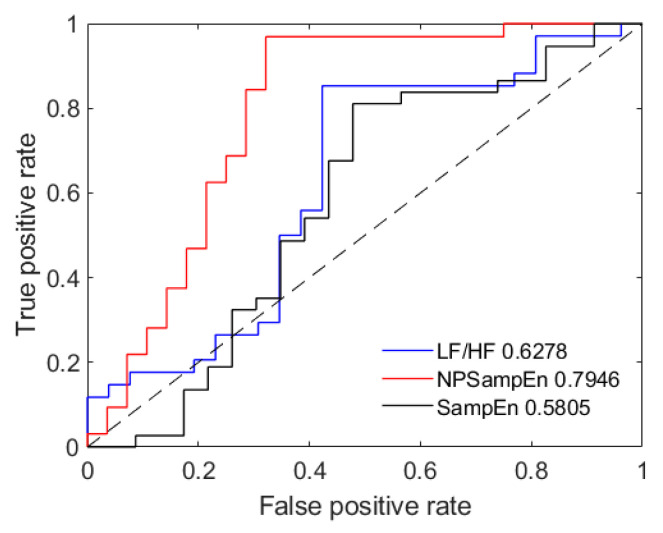
The receiver operating characteristic (ROC) curves of LF/HF, NPSampEn, and SampEn.

Samples used during the OSA recording screening were obtained by calculating the mean of the segments for each recording. Fisher’s discriminant function (SPSS) was used to classify each sample as normal or apneic. The accuracy (ACC), sensitivity (SEN), specificity (SPE), and area under the receiver operating characteristic curve (AUC) values of SampEn, NPSampEn, and LF/HF were computed and are displayed in Table 2. The ACC was defined as the percentage of correctly classified samples. The SEN was defined as the percentage of correctly classified OSA samples. The SPE was defined as the percentage of correctly classified healthy samples. The AUC reflected the recall ratio of the indices. As shown in Table 2, for 60 samples, NPSampEn achieved the highest ACC (83.3%), SEN (77.5%), and SPE (95%) among these indices, which were higher than those of SampEn and LF/HF. Moreover, Figure 7 shows the receiver operating characteristic (ROC) curve and area under the ROC curve (AUC) of SampEn, NPSampEn, and LF/HF. The closer the curve is to the upper left corner, the larger the area under the curve, and the higher the recall ratio of the index. As shown in Figure 7, NPSampEn achieved the highest AUC value (0.795), and SampEn and LF/HF yielded lower AUC values (0.581 and 0.628, respectively). Therefore, NPSampEn had the highest recall ratio.

Taking these results into consideration, it was indisputable that NPSampEn improved the OSA screening accuracy, and the classification results showed that NPSampEn is a more reliable index for ruling out OSA compared with SampEn and LF/HF.

## 4. Discussion

### 4.1. Comparison and Summary

The purpose of the study was to identify a useful single index that has interpretability and can improve the recognition performance of a classifier. Among the classic indices, the LF/HF had great OSA detection performance (Table 2), which is consistent with the results in other studies [12,13,26]. Moreover, NPSampEn yielded high screening performance and enabled the quantification of OSA severity (Figure 4, Table 2). The simulation results also proved that NPSampEn is more suitable than the other indices for short-term complexity analysis (Figure 3). Therefore, NPSampEn is more suitable for the short-term assessment of autonomic nerves and can eliminate the influence of individual differences and short-term fluctuations.

There are some previous works using the same dataset to detect OSA, although the number of features and lengths of RR segment were not completely consistent [16,26,34,35,36]. As can be seen in Table 3, some of the studies needing longer RR segments showed the performance of their introduced methods [26,34,35]. Reference notes are usually created every minute to indicate whether apnea occurred in that minute. The 1-min RR segments had better real-time performance and are more suitable for OSA detection compared with 1000-second and 5-min RR segments [37,38]. In a previous study, Al-Angari et al. analyzed the 1-min RR segment and calculated the SampEn index and frequency domain index of each minute segment. The results showed that the HRV analysis of 1-min RR segments was effective to detect OSA [16]. Some of the other studies also used 1-min RR segments to detect OSA, but these were based on multi-feature and achieved lower accuracy than that of our proposed method [16,36]. Our method not only guarantees the real-time performance, but also achieved high accuracy.

### 4.2. Method Motivation Analysis

In previous studies, the LF/HF has been considered as a useful linear tool for OSA screening; however, it is not suitable for analyzing nonlinear and nonstationary systems [14,15]. Entropy methods have been widely used to identify subtle changes and complexities in HRV analysis. Classic nonlinear analysis methods including SampEn and fuzzy entropy have some advantages, but there are some limitations. On one hand, reference annotations are usually made for every minute with 60–100 RR intervals to determine the presence or absence of apnea during the corresponding minute [21]. Nevertheless, SampEn and fuzzy entropy have limitations in short-term HRV analysis for two reasons. The values of SampEn and fuzzy entropy are undefined when inappropriate parameters are selected such as an inappropriate r parameter. In addition, the values of SampEn and fuzzy entropy may not accurately reflect the complexity of the signal (Figure 3f). On the other hand, the SampEn and fuzzy entropy values depend on the selection of the parameter r, which is closely related to the standard deviation of the sequence. Nevertheless, the standard deviation can only reflect the fluctuation of the sequence integrally; it cannot fully reflect the subtle fluctuations when quantizing the template distance. In addition, r values that are too similar will lead to unnecessary redundancy in the values, and increase the computational expense, while this problem occurs with fuzzy entropy due to distance quantization. Therefore, NPSampEn was proposed for use in short-term data analysis.

The results showed that NPSampEn (83.3%) significantly improved the OSA screening performance compared to SampEn (68.3%) and LF/HF (73.3%), and successfully quantified the severity of OSA (*p* < 0.05). Instead of the constant r value, the r solution of NPSampEn is defined based on the distances between the template vectors. The cumulative probability of the template distance determined by the set of r values can more accurately and precisely quantify the distribution of template distances in each region, and describe the signal fluctuation more specifically. The advantages of NPSampEn include its sensitivity to subtle fluctuations in signals, as the set of r values are defined by the unique template distance. As they constitute a set of adaptive values, the set of r values depends more on the dynamics of the signal and is less affected by the signal length. Therefore, NPSampEn is an adaptive method that is suitable for short-term sequences and fully reflects the fluctuations of sequences; furthermore, it can be used to overcome the influence of individual differences and identify the subtle fluctuations in physiological signals.

### 4.3. Physiological Significance

OSA affects the balance of the autonomic nervous system (ANS), thus affecting HRV [8]. In this study, increased LF/HF values were observed in OSA patients (Table 1, Figure 4a). This result indicates that the sympathetic nerve is more active in OSA patients. A number of mechanisms may be responsible for this finding. Relative hypoxemia may act on central chemoreceptors to increase the sympathetic tone [12].

The regulation of the autonomic nerve to the heart rate is a nonlinear and nonstationary process, and short-term sequence analysis has a considerable advantage in overcoming nonstationary interference [39]. Therefore, NPSampEn, a short-term data analysis method, is suitable for analyzing the complexity of nonstationary systems, which reflect disruptions of the ANS.

The ANS disorder caused by OSA events is related to individual differences [40]; therefore, it is not appropriate to use the classical time-frequency domain indices for OSA screening and quantification. In the calculation of NPSampEn, the adaptive parameter r is determined according to the physiological signals from different individuals, which can effectively avoid the influence of individual differences to better estimate the regularity of the physiological signals. Therefore, NPSampEn provides a new method for the analysis of HRV in the ANS of OSA patients.

### 4.4. Limitations

There are some limitations in the present study. First, the approach could face the problem in a more robust and reliable way and should be done on a larger set of recordings. More databases should be used to validate the effect of the method in future study. Second, it should define a training, validation, and testing framework in order to have comparable performance measures with the literature in future study.

## 5. Conclusions

In this study, nonparametric sample entropy (NPSampEn) was proposed as a nonlinear index to evaluate the heart rate variability and complexity of the autonomic nervous system (ANS) in OSA patients. The results showed that the ANS changes were significantly different between the normal and OSA groups. The results also showed that the complexity of ANS decreased significantly as the severity of OSA increased. Therefore, the study in this paper basically proves that NPSampEn is extremely suitable for OSA short-term detection and performs well in autonomic nerve complexity analysis.

## Figures and Tables

**Table 1:** Entropy/frequency domain indices for the normal, mild-moderate, and severe obstructive sleep apnea (OSA) groups.

	Indices	N (Mean ± SD)	MOSA (Mean ± SD)	SOSA (Mean ± SD)	*p* Value		
					N & MOSA	N & SOSA	MOSA & SOSA
Entropy	SampEn	0.587 ± 0.058	0.564 ± 0.094	0.443 ± 0.097	0.426	0 ***	0 ***
	NPSampEn	0.270 ± 0.014	0.243 ± 0.039	0.199 ± 0.029	0.006 **	0 ***	0 ***
Frequency domain	LF	0.0060 ± 0.0075	0.0010 ± 0.0007	0.022 ± 0.054	0.687	0.150	0.088
	HF	0.0050 ± 0.0078	0.00047 ± 0.00042	0.0074 ± 0.022	0.404	0.608	0.184
	LF/HF	2.049 ± 0.717	3.785 ± 2.000	4.975 ± 2.437	0.012 *	0 ***	0.067

*, **, *** represent *p* < 0.05, *p* < 0.01, and *p* < 0.001, respectively

**Table 2:** Performance comparisons of different indices for obstructive sleep apnea (OSA) screening.

	Indices	TP	TN	FP	FN	ACC	SEN	SPE	AUC
Nonlinear indices	SampEn	29	12	8	11	68.3%	72.5%	60.0%	0.581
	NPSampEn	31	19	1	9	83.3%	77.5%	95.0%	0.795
Frequency domain	LF/HF	29	15	5	11	73.3%	72.5%	75%	0.628

NPSampEn: non-parametric sample entropy; LF/HF: ratio of low frequency power to high frequency power. TP: true positive. TN: true negative; FP: false positive; FN: false negative; ACC: accuracy; SEN: sensitivity; SPE: specificity; AUC: area under the receiver operating character curve.

**Table 3:** Comparison of the classification results between our proposed method and previous works on the Physionet database.

Reference	Method	Features	Length of RR Segment	Results
Pietrzak et al. [34]	Standard deviation of successive difference	single feature	1000 s	ACC = 88.5% SEN = 96.0% SPE = 70.0%
Ravelo-García et al. [35]	Permutation entropy	multi-feature	5 min	ACC = 78.0% SEN = 64.3% SPE = 86.5%
Li et al. [26]	Variance delay fuzzy approximate entropy	single feature	5 min	ACC = 90.0% SEN = 87.5% SPE = 95.0%
Al-Angari et al. [16]	Sample entropy *m* = 1, 2, 3	multi-feature	1 min	ACC = 70.3% SEN = 69.5% SPE = 70.8%
Varon et al. [36]	support vector machine	multi-feature	1 min	ACC = 84.7% SEN = 84.7% SPE = 84.7%
Proposed method	Nonparametric sample entropy	single feature	1 min	ACC = 83.3% SEN = 77.5% SPE = 95.0%

## Data Availability

Not applicable.
